# The Role of Neuroinflammation in Postoperative Cognitive Dysfunction: Moving From Hypothesis to Treatment

**DOI:** 10.3389/fpsyt.2018.00752

**Published:** 2019-01-17

**Authors:** Seyed A. Safavynia, Peter A. Goldstein

**Affiliations:** ^1^Department of Anesthesiology, Weill Cornell Medical College, New York, NY, United States; ^2^Department of Medicine, Weill Cornell Medical College, New York, NY, United States; ^3^Neuroscience Graduate Program, Weill Cornell Medical College, New York, NY, United States

**Keywords:** postoperative cognitive dysfunction, cognitive decline, neuroinflammation, central nervous system, microglia, anesthesia

## Abstract

Postoperative cognitive dysfunction (POCD) is a common complication of the surgical experience and is common in the elderly and patients with preexisting neurocognitive disorders. Animal and human studies suggest that neuroinflammation from either surgery or anesthesia is a major contributor to the development of POCD. Moreover, a large and growing body of literature has focused on identifying potential risk factors for the development of POCD, as well as identifying candidate treatments based on the neuroinflammatory hypothesis. However, variability in animal models and clinical cohorts makes it difficult to interpret the results of such studies, and represents a barrier for the development of treatment options for POCD. Here, we present a broad topical review of the literature supporting the role of neuroinflammation in POCD. We provide an overview of the cellular and molecular mechanisms underlying the pathogenesis of POCD from pre-clinical and human studies. We offer a brief discussion of the ongoing debate on the root cause of POCD. We conclude with a list of current and hypothesized treatments for POCD, with a focus on recent and current human randomized clinical trials.

## Introduction

Disordered neurocognitive function following surgery is a heterogenous set of conditions, which includes both the fluctuating and typically transient postoperative delirium and the more protracted problem of postoperative cognitive dysfunction (POCD). POCD is a well-known risk of the surgical experience, having been described as a consequence of anesthesia as early as 1887 (1), and a common complication of cardiac surgery since the 1950's (2). More than 60 years following its modern description, it is only just now that clearly articulated guidelines have been suggested for identifying POCD (3). POCD has been loosely defined as a significant reduction in cognitive performance from baseline following surgery, and diagnosed as subtle deficits in multiple core neurocognitive domains, including executive function, attention, verbal memory, psychomotor speed, and visuospatial abstraction (4, 5). Given that the literature thus far has used the term POCD to describe these deficits, we will also use the term here, but recognize going forward the nomenclature will likely evolve so as to conform with new guidelines (3). Since the 1950's, advanced age has been shown to be one of the strongest associations for development of POCD: the incidence of POCD is reported to be anywhere between 9 and 54% 1 week after surgery in adults over age 65 (6), with no difference in rates based on the type of surgery and/or anesthetic (7). POCD itself can persist long after surgery, with an incidence between 10 and 17% at 3 months following surgery (7, 8) and 3% at 12 months following surgery (9). Moreover, POCD can contribute to severe cognitive deficits over the long term, affecting overall morbidity and mortality, with increased hospital costs (10, 11). The health and economic burdens of POCD are likely to increase over the next several years: Life expectancy is increasing, and more than 30% of individuals over age 65 have surgery annually (12).

At the epidemiological level, a handful of risk factors for the development of POCD have emerged from population studies; controversy exists, however, in the interpretation of these data and their clinical implications. Risk factors for POCD were initially identified in patients undergoing cardiac surgery, and included advanced age, aortic valve replacement, and prolonged (mean 70 min) cardiopulmonary bypass (CPB) time (13). While advanced age (>65 years) has been consistently identified as a risk factor for POCD (8, 14), the evidence is less convincing with other potential risk factors due to differences in populations and neurocognitive testing modalities (4, 7). For example, it has long been thought that preexisting frailty in general (15–20), and neurocognitive frailty in particular (9, 21, 22) may be a risk factor for POCD as these patients may be vulnerable to cognitive insults at baseline. Indeed, observational studies have shown that surgery may precipitate further cognitive decline in patients with neurodegenerative disorders such as Alzheimer's disease (AD) (23), and biomarkers of AD such as the apolipoprotein E4 (APOE-4) genotype have also been associated with development of POCD in elderly patients (24, 25). However, a long-term retrospective analysis did not show an accelerated progression to dementia in patients with AD after non-cardiac surgery (26). More recent data in humans show that while the CSF tau/β-amyloid ratio increases following surgery, the increase is independent of the type of anesthetic (i.e., propofol vs. isoflurane) (27), further calling into question the predictive value of these biomarker studies. These discrepancies may be in part due to confounders such as temperature regulation; hypothermia rather than anesthesia *per se* seems to be the driver behind the observed tauopathy (28, 29), with dexmedetomidine as a possible exception (30). Chronic inflammatory states such as diabetes, metabolic syndrome, and atherosclerosis have all been proposed as potential risk factors for POCD (31–33), while pro-cognitive activities such as sleep, exercise, and level of education seem to be protective (34). Despite these data, the heterogeneous populations and study paradigms used inherently limit the clinical interpretation of these risk factors.

At the cellular level, data from animal and human studies suggest that neuroinflammation from either surgery or anesthesia is a major contributor to the development of POCD, yet the specific relationship between inflammation and POCD remains unknown. Multiple rodent models of surgery have shown upregulation of pro-inflammatory cytokines and inflammatory mediators in both peripheral tissues and the central nervous system (CNS) (35, 36). Similarly in rats, inflammation in the form of prior infection can also increase the incidence and severity of POCD (37, 38). In human studies, patients who develop POCD also show increases in serum and cerebrospinal fluid (CSF) pro-inflammatory cytokines, irrespective of the type of surgery (39–42), which has been corroborated in meta-analyses (43, 44). However, there seems to be little relationship between the magnitude of the neuroinflammation and the development of POCD. For example, while CPB was thought to be a strong initiator of peripheral and subsequent neuroinflammation (45), the rates of POCD in cardiac and non-cardiac surgery are similar (7), as well as in pulsatile vs. non-pulsatile CPB (46) and on-pump and off-pump cardiac surgery (45). Meta-regressions show a slight relationship with plasma levels of interleukin-6 (IL-6) and S100 calcium-binding protein β (S100β) and POCD, but no other cytokines studied have shown any correlation (43). While inflammation always occurs with surgery, POCD does not, and it remains unclear what specific risk factors and triggers are responsible for this conversion.

Despite the advances in research, fundamental barriers exist to understanding POCD in a generalized context, limiting the ability to predict patients at risk for POCD and develop appropriate therapies for such patients. Firstly, POCD has been broadly defined, with no historical formal clinical definition (5, 47, 48). Similarly, animal models of POCD are defined using a variety of metrics, each testing different cognitive domains as a proxy for POCD (49). Without a formal definition, it is difficult to accurately and consistently identify patients with POCD and construct appropriate animal models, thereby limiting a generalized understanding of the epidemiology and pathogenesis of the disorder. Secondly, determining the root causes of POCD is difficult as surgery and anesthesia occur almost invariably in tandem (48), with larger and more high-risk surgery often necessitating longer anesthetic times. Thirdly, proposed treatments showing promise in animal studies are often not as effective when tested in clinical trials, revealing a need for a more nuanced understanding of POCD.

We present a broad topical overview of the current state of the literature regarding the effects of neuroinflammation on the development of POCD. We will review the proposed cellular mechanisms underlying the pathogenesis of POCD in pre-clinical and human studies. We will present the evidence underlying the debate on the etiologic contributions of neuroinflammation and POCD in both animal models and human studies, whether surgical, anesthetic, or both. Lastly, we will discuss proposed treatments for POCD, with a focus on recent and current human randomized clinical trials.

While POCD is often grouped with postoperative delirium (POD) in the literature, we limit the discussion in this review to POCD and not POD. POD and POCD are distinct disorders: Delirium is defined in the fifth edition of the Diagnostic and Statistical Manual of Mental Disorders (DSM-5) as a disorder of reduced attention and orientation to the environment, accompanied by cognitive disturbances in an acute and fluctuating course with lucid intervals (50). By contrast, POCD is described as an objectively measured decline in cognition in the postoperative state compared to the preoperative state (48). Unlike delirium, the time course of POCD does not fluctuate with lucid intervals, and some patients never recover from the initial insult (51, 52). Nevertheless, there is a growing body of evidence suggesting that neuroinflammation contributes to POD; for a detailed review on the role of neuroinflammation on POD, please see Maldonado (53).

## Proposed Mechanisms for Pathogenesis of POCD

Taken together, data from animal and human studies have fueled the ***hypothesis*** *that peripheral surgical trauma causes CNS inflammation via disruption of the blood-brain barrier (BBB), which then causes a functional disruption in neural activity, leading to POCD*. Each component of this hypothesis is regulated by a variety of inflammatory mediators discussed below. This sequence of events can persist long after surgery and resolution of neuroinflammation, and can accelerate neurocognitive decline in neurocognitively frail populations.

### Peripheral Initiation of Inflammation

**Figure 1:**
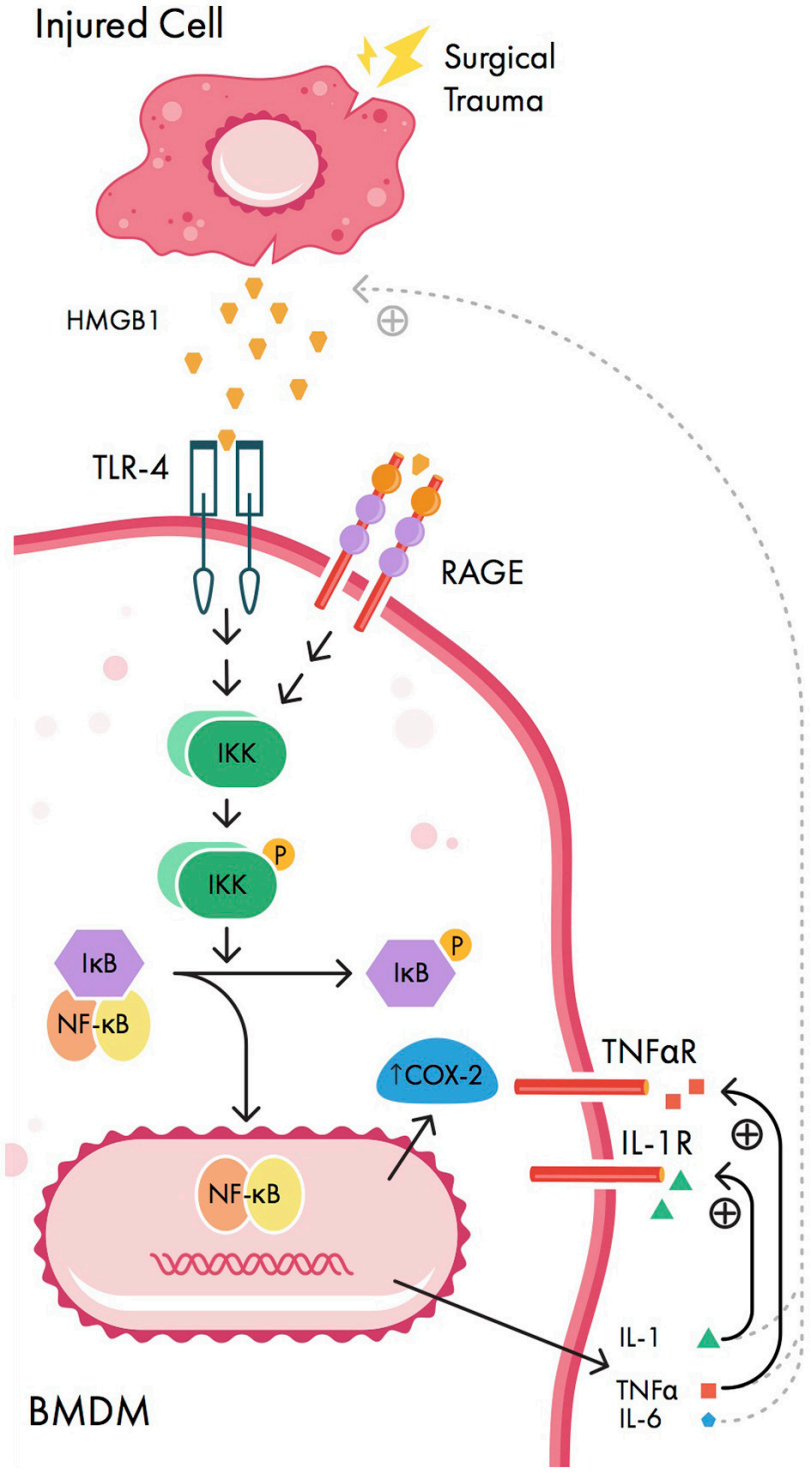
Signaling pathways involved in peripheral initiation of inflammation. Injured cells release damage-associated molecular patterns (DAMPs) including high mobility group box-1 protein (HMGB1) in response to surgical trauma. HMGB1 activates nuclear factor-kappa B (NF-κB) signaling pathways in bone marrow derived monocytes (BMDMs), causing nuclear translocation of NF-κB, increased expression of cyclooxygenase 2 isozyme (COX-2) upregulation, and expression of pro-inflammatory cytokines interleukin-1 beta (IL-1β), interleukin 6 (IL-6), and tumor necrosis factor alpha (TNFα). These pro-inflammatory cytokines can act back on BMDMs in positive feedback loops (solid curved lines) as well as promote further release of HMGB1 from injured cells by unknown mechanisms (dashed curved lines). IKK, IκB kinase; IL-6R, IL-6 receptor; P, phosphate group; RAGE, receptor for advanced glycosylation end products; TLR-4, Toll-like receptor 4; TNFαR, TNFα receptor.

It is well-known that aseptic surgical trauma causes inflammation at the surgical site, which is amplified *via* peripheral pro-inflammatory cytokines. In response to surgical trauma, damaged cells at the site of injury passively release small biomolecules known as damage-associated molecular patterns (or danger-associated molecular patterns; DAMPs) (4, 54). In particular, the DAMP known as high molecular group box 1 protein (HMGB1) is released following surgical trauma and binds to Toll-like receptors (TLRs) and the receptor for advanced glycosylation end products (RAGE) on the cell membrane of peripherally circulating bone marrow derived monocytes (BMDMs) (55) (Figure 1). In rats, surgery and anesthesia have been associated with increased hippocampal HMGB1 expression (56); similarly, human studies have shown that plasma HMGB1 levels are correlated with the level of inflammation in both non-cardiac surgery and non-surgical inflammatory states (57). In rodents, elevations of HMGB1 are associated with cognitive deficits (58), which can be mitigated in the presence of HMGB1 inhibitors (4, 59). These results are corroborated by evidence that HMGB1 levels are elevated in patients with POCD following gastrointestinal surgery (60).

When bound by HMGB1, both TLR-4 and RAGE activate nuclear factor kappa B (NF-κB), a transcription factor which regulates the expression of pro-inflammatory cytokines (Figure 1). Normally, cytosolic NF-κB is bound to the NF-κB inhibitor IκB in an inactive state; however, when IκB is phosphorylated by IκB kinase (IKK), NF-κB is released and enters the nucleus, causing pro-inflammatory cytokine upregulation (55). Once activated by NF-κB, the pro-inflammatory cytokines interleukin-1 beta (IL-1β), IL-6, and tumor necrosis factor alpha (TNFα) cause further release of HMGB1 in a positive feedback loop, amplifying the inflammatory response (57). Additionally, IL-1 and TNFα can cause further activation of NF-κB, resulting in cyclooxygenase 2 isozyme (COX-2) upregulation (34). There is a strong association between elevations in serum pro-inflammatory cytokines and POCD in both animal models (61, 62) and human studies (41, 44). Moreover, in rats, inhibition of NF-κB and pro-inflammatory cytokines has been associated with a reduction in POCD using various metrics (including Morris water maze, elevated plus maze, fear conditioning, and passive avoidance test) (63–65).

### Blood-Brain Barrier Breakdown

**Figure 2:**
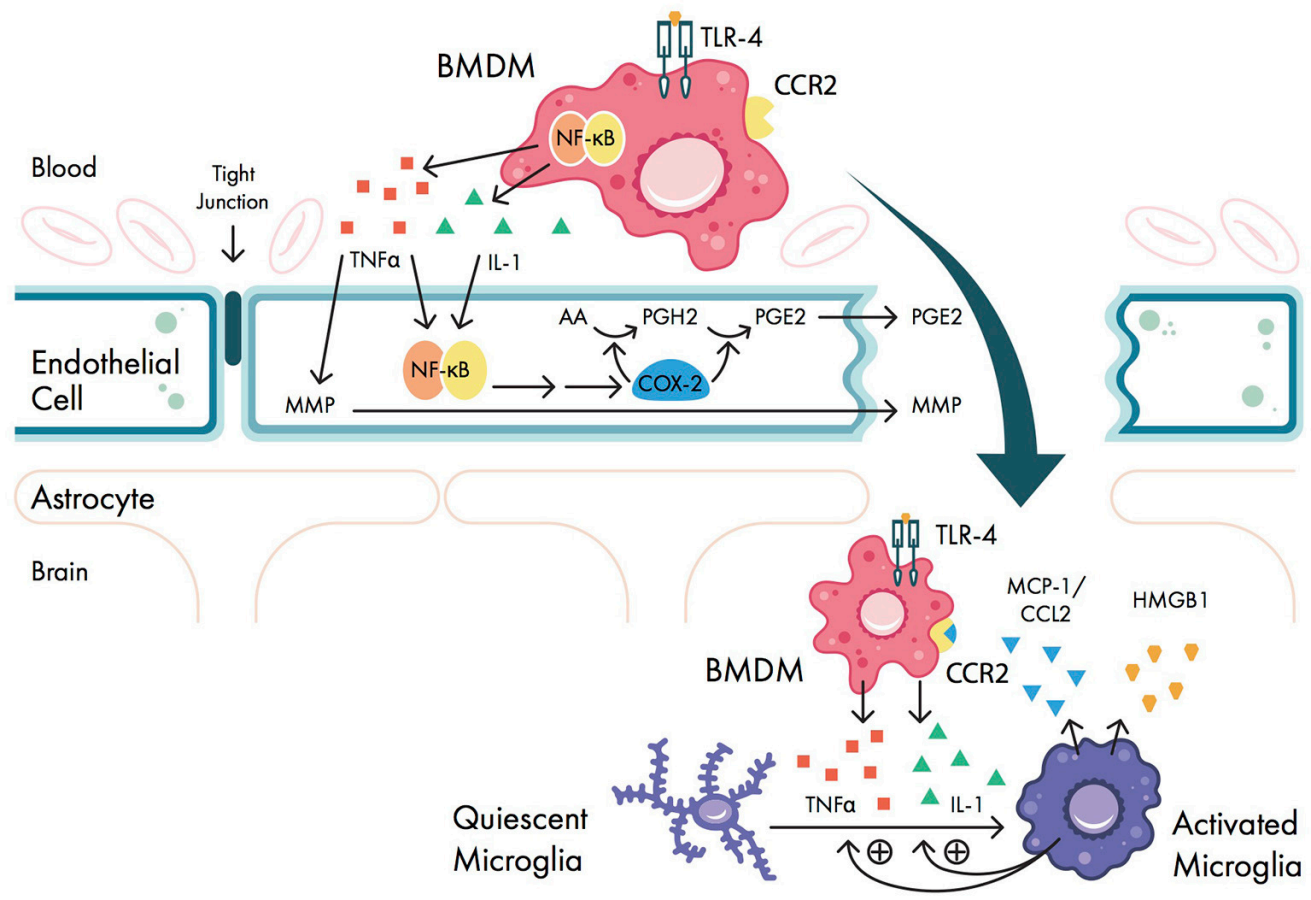
Signaling pathways involved in blood-brain barrier (BBB) breakdown. Pro-inflammatory cytokines interleukin-1 (IL-1) and tumor necrosis factor alpha (TNFα) are secreted by bone marrow derived monocytes (BMDMs) and cause upregulation of nuclear factor-kappa B (NF-κB) and matrix metalloproteinase (MMP) expression in vascular endothelial cells. NF-κB activation causes downstream upregulation of cyclooxygenase 2 isozyme (COX-2) expression, which promotes prostaglandin synthesis and disrupts BBB permeability. Once the BBB is disrupted, BMDMs can enter the central nervous system (CNS); here, the pro-inflammatory cytokines IL-1 and TNFα promote the activation of quiescent microglia. These microglia promote further release of IL-1 and TNFα from BMDMs, as well as secrete high mobility group box-1 protein (HMGB1) and the chemokine monocyte chemo-attractant protein 1 (MCP-1, also called C-C motif ligand 2 (CCL2)). MCP-1/CCL2 binds to the BMDM cell surface receptor chemokine receptor type 2 (CCR2), further promoting BMDM migration into the CNS. AA, arachidonic acid; PGE2, prostaglandin E2; PGH2, prostaglandin H2; TLR-4, Toll-like receptor 4.

Peripheral pro-inflammatory cytokines disrupt BBB permeability *via* COX-2 upregulation and matrix metalloproteinases (MMPs), allowing pro-inflammatory cytokines to enter the CNS (Figure 2). Normally, the BBB is made up of tight junctions held together by transmembrane proteins (i.e., occludins, claudins, junctional adhesion molecules) between neurovascular endothelial cells (66). This structure only allows for the passive diffusion of water, gases, and small lipid-soluble molecules (67). However, pro-inflammatory cytokines IL-1 and TNFα can upregulate COX-2 in neurovascular endothelial cells, which promotes local prostaglandin synthesis (68) and disrupts BBB permeability (69) (Figure 2). TNFα, IL-1β, and IL-6 have all been found in hippocampal tissue in rats (69–71) and in human CSF (42, 72) following surgical trauma, suggestive of a breakdown in the BBB. Cytokine elevation in the CNS has also been associated with memory dysfunction in mice (73) and cognitive dysfunction (measured by different neurocognitive metrics—see Table 2) in humans (41, 42). These data suggest that BBB breakdown is associated with cytokine influx and cognitive impairment, however this evidence does not rule out the possibility that the cytokine elevation may be generated locally within the CNS. More convincingly, immunoglobulin G (IgG), which is not present normally in the brain, has also been identified in hippocampal slices in rats following surgery (56, 74). Similarly, CNS-specific proteins such as S100β and neuron-specific enolase (NSE) are found in plasma following cardiac and non-cardiac surgery in patients with POCD (43, 75, 76). TNFα can also upregulate transcription of MMPs, particularly MMP-9; this aberrant MMP expression can degrade extracellular matrix proteins *in vitro*, further breaking down the BBB (66) (Figure 2). Unfortunately, there is only limited *in vivo* evidence concerning the role of MMPs in BBB disruption (66). At a functional level however, MMP-9 gene deletion mice exposed to surgical trauma have been shown to exhibit better cognitive performance (in terms of fear conditioning) compared to wild-type mice (77).

Lastly, once the BBB is disrupted, circulating BMDMs in the periphery are able to enter the CNS and augment neuroinflammation *via* cytokine expression and microglial activation (Figure 2). While mast cells and microglia exist in the CNS, there are no normally occurring populations of dendritic cells or monocytes (78). In the setting of inflammation and BBB breakdown however, BMDMs are recruited to the CNS (79) *via* interactions between the chemokine monocyte chemo-attractant protein 1 [MCP-1, also called C-C motif ligand 2 (CCL2)] and the BMDM cell surface receptor chemokine receptor type 2 (CCR2) (Figure 2). Once the BMDMs are present in the CNS, they continue to secrete pro-inflammatory cytokines *via* upregulation of NF-κB transcription (34), and activate microglia in the CNS, further amplifying the neuroinflammation. In mice it has been shown that preoperative depletion of BMDMs reduced POCD (80), suggesting that BMDM migration plays a pivotal role in POCD. Taken together, once the BBB is disrupted, cytokines can freely enter the CNS, causing trafficking of BMDMs to neural tissues and initiating poorly regulated immune functions.

### Microglial Activation

Microglia are known as the “resident macrophages” of the CNS (81) and have many important contributory functions in the CNS, including synaptic pruning during development (82) and synaptic scaling in neural plasticity (83). Derived from yolk-sac cells, microglia migrate to the CNS early in development, before the differentiation of many cell types in the CNS (81). As a part of the innate immune system, microglia surveil brain parenchyma (84) and are the first responders to pathogens in the CNS. Although a fully differentiated cell, microglia have the unique ability to self-replenish within the CNS (85).

Normally, microglia are in an inactive state maintained by binding of the CX3CR1 protein to the microglial CX3CR1 receptor (86). However, in the setting of inflammation and BBB breakdown, they can differentiate into one of two activated phenotypes, M1 and M2 (87). The M1 phenotype has high phagocytic properties and is pro-inflammatory (88, 89), while the M2 phenotype is involved in tissue repair and remodeling and is anti-inflammatory (90). Not surprisingly, pro-inflammatory mediators such as TNFα or lipopolysaccharide promote microglial differentiation into the M1 phenotype (91). Moreover, TNFα blockade can suppress microglial activation in mice (35). Conversely, anti-inflammatory cytokines such as IL-4 are known to play a role in promoting the alternative M2 phenotype (88). However, recent evidence is beginning to challenge the dichotomy of the M1/M2 phenotypes, suggesting that there are many overlapping phenotypes with various functions and activation pathways (92). One such new area is the role of mast-cell degeneration in activating microglia: In a recent rat study, Zhang et al. (93) showed that peripheral surgery induced CNS mast cell degranulation and subsequent microglial activation. Further, administration of cromolyn sodium (which inhibits mast cell degranulation) inhibits microglial activation in rats (93, 94), demonstrating a new microglial interaction and a possible new therapeutic target for POCD.

Once microglia are activated, they continue to upregulate expression of pro-inflammatory cytokines, thus amplifying neuroinflammation and contributing to the development of POCD (Figure 2). Activated microglia are known to release HMGB1, TNFα, and IL-1β in a variety of rodent models (95–97). Further, astrocytes and microglia both upregulate expression of MCP-1/CCL2 (98), and astrocyte CCL2 can induce further microglial activation *in vitro* (99, 100). These chemokines cause further influx of BMDMs into the CNS: Trafficked BMDMs in turn can activate microglia to the M1 phenotype *via* TNFα/IL-1 expression, and activated microglia recruit more BMDMs into the CNS *via* reciprocal TNFα expression (101). In aged mice, microglial activation is increased in POCD (37, 49, 102). Moreover, in mice, both perioperative microglial depletion (103) and promotion of an M2 phenotype *via* erythropoietin administration (99) improved memory dysfunction as measured by passive avoidance and novel object recognition tests.

### The Role of Oxidative Stress

In addition to the inflammatory pathways described above, surgical trauma can also produce oxidative stress and deplete the body of antioxidants (57); these oxidative processes, when superimposed on the inflammatory pathway, can contribute to the development of POCD. Surgical stimulation in rodents can raise the levels of CNS nicotinamide adenine dinucleotide phosphate (NADPH) oxidase, an enzyme compound that generates superoxide in response to stress (104). The superoxide radicals in turn generate other reactive oxygen species (ROS), potentially causing direct damage of neural tissues. Additionally, peripheral oxidative stress can also disrupt the BBB (105), representing a convergence of oxidative stress with the neuroinflammatory pathway. Within the CNS, microglia have been shown to release ROS (106) in response to both HMGB1 (107) and S100β (108). Of note, activated microglia are known to release HMGB1 (97), creating the opportunity for yet another neuroinflammatory positive feedback loop.

Recent evidence from animal and human studies suggests that oxidative stress alone can contribute to POCD. Hippocampal neurons are very metabolically active and are some of the most sensitive neurons to oxidative stress (109); it follows that hippocampal injury from oxidative stress can have profound effects on memory formation and spatial navigation. In aged rats, tibial fracture surgery was associated with memory impairments (measured by open field task and novel object recognition task) on postoperative day 1 with corresponding increases in oxidative damage in the hippocampus and prefrontal cortex (109). Oxidative injury from hypoglycemia has also been shown to induce cognitive impairment in rats, and inhibition of NADPH oxidase has been shown to mitigate such impairments (110). In humans, levels of the ROS nitric oxide are correlated with development of POCD (*via* neurocognitive battery) at 4 days and 3 months following cardiac surgery (111).

### Functional Consequences of CNS Inflammation

Memory formation occurs in the hippocampus and is achieved by a process known as long-term potentiation (LTP). Although the mechanisms of induction and maintenance of LTP at various synapses in the CNS are very complex and somewhat controversial, LTP is thought to be achieved by high frequency glutamatergic activation of hippocampal neurons (112). At rest, presynaptic glutamatergic Schaffer cells signal to post-synaptic CA1 collateral neurons. The CA1 neurons themselves contain three types of glutamate receptors: the metabotropic Glu2 receptor and the ionotropic AMPA and NMDA receptors. During normal, low-frequency stimulation of CA1 neurons, glutamate acts on all receptors, but the NMDA channels are blocked by magnesium. With high frequency stimulation however, postsynaptic depolarization causes an activation of NMDA receptors, which causes an influx of calcium and activation of second messenger systems (112). Downstream, the number and sensitivity of AMPA receptors is increased through phosphorylation, and synaptic strength is increased, resulting in memory formation (113).

The presence of pro-inflammatory cytokines can have detrimental effects on the regulation of neurotransmitter signaling in the hippocampus, ultimately resulting in excitotoxic neuronal damage and resulting cognitive impairment. First, the hippocampus has a large number of cytokine receptors, rendering it susceptible to high concentrations of pro-inflammatory cytokines such as IL-1 and TNFα in neuroinflammatory processes (114, 115). Once these cytokine receptors are activated at high levels, there is a downregulation of metabotropic Glu2 receptors causing enhanced AMPA/NMDA signaling, disrupting the process of LTP (116). Meanwhile, HMGB1 can also potentiate glutamate signaling through NMDA, causing an increased influx of glutamate in hippocampal neurons, which ultimately results in glutamate toxicity (117). Further, TNFα can depress inhibitory neurotransmission *via* downregulation of GABA receptors, disrupting the delicate balance of excitatory and inhibitory neurotransmission and ultimately favoring glutamate toxicity (118). These detrimental effects are compounded by the T-cell mediated release of glutamate from activated microglia *via* a separate glutamate transporter subtype (119). Collectively, the aforementioned mechanisms contribute to glutamate toxicity in the hippocampus, resulting in neuronal death and cognitive impairment.

### Cholinergic Anti-inflammatory Pathway

**Figure 3:**
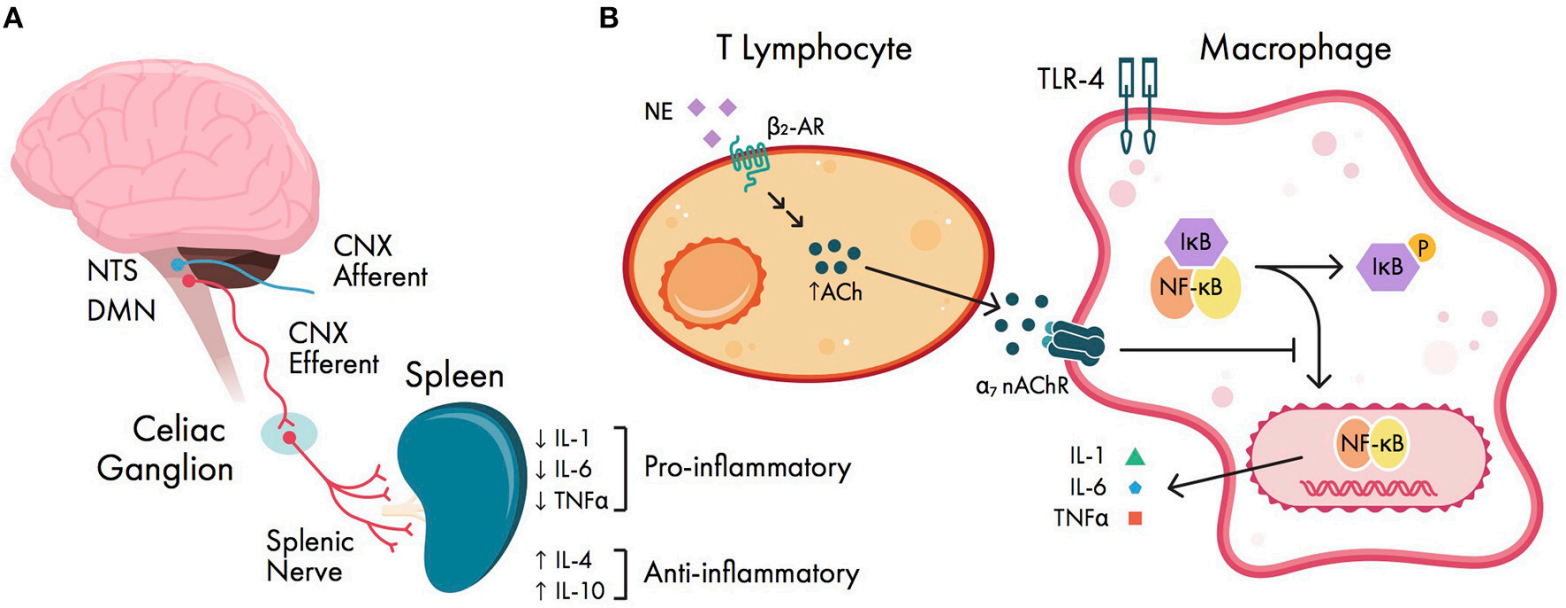
Cholinergic anti-inflammatory pathway. **(A)** schema of vagal reflex arc. Damage-associated molecular patterns (DAMPs) are sensed by vagal afferents; the efferent vagal arc terminates in the celiac ganglion onto splenic nerve fibers, ultimately causing downregulation of pro-inflammatory cytokines and upregulation of anti-inflammatory cytokines. **(B)** cellular signaling within the cholinergic anti-inflammatory pathway. Splenic nerve endings terminate near T lymphocytes and increase acetylcholine (ACh) production *via* β_2_ adrenergic receptors (β_2_-ARs). The expressed ACh can activate circulating macrophages *via* alpha-7 nicotinic ACh receptors (α7 nAChRs). Activation of α7 nAChRs causes downstream inhibition of NF-kB activation, ultimately decreasing pro-inflammatory cytokine release. CNX, cranial nerve X (vagus nerve); DMN, dorsal motor nucleus of the vagus; IL, interleukin; NE, norepinephrine; NTS, nucleus tractus solitarius; P, phosphate group; TLR-4, Toll-like receptor 4; TNFα, tumor necrosis factor alpha.

Although peripheral pro-inflammatory cytokines are the primary initiator of neuroinflammation, they are also involved in regulating the inflammatory response *via* a vagal reflex arc (34) (Figure 3). This serves to help limit the degree of inflammation and protect organ systems from further damage. In this arc, DAMPs released from surgical trauma are sensed by vagal afferents that terminate on the nucleus tractus solitarius (NTS) (120). The efferent arc of this reflex originates from fibers within the dorsal motor nucleus of the vagus, sending signals to the celiac ganglion. Within the celiac ganglion, vagal efferents regulate postganglionic catecholaminergic fibers *via* functional connections within the splenic nerve (121). The splenic nerve endings are in close anatomical position with T lymphocytes, which express β_2_ adrenergic receptors (122). When activated, T lymphocytes upregulate transcription of choline acetyltransferase, facilitating synthesis of acetylcholine (ACh) (120); this newly synthesized ACh can then activate circulating macrophages that express alpha-7 nicotinic ACh receptors (α7 nAChRs). Ultimately, activation of α7 nAChR-expressing macrophages causes inactivation of NF-kB, which decreases cytokine release (34). In addition, vagal stimulation is known to induce regulatory T-cells and secretion of anti-inflammatory cytokines IL-4 (which promotes microglial differentiation to the M2 phenotype) and IL-10 (123, 124). One experiment in rats treated with the cholinesterase inhibitor physostigmine following laparotomy showed a reduction in hippocampal IL-1β and TNF α expression and hippocampal damage (125). In humans, anticholinergic drugs are widely known to precipitate POCD (126), although it is unclear whether the cholinergic anti-inflammatory pathway is involved in this process. Thus, it has been proposed that vagal stimulation may mitigate the development of POCD (127), although this remains untested in human literature.

The vagus nerve also regulates pro-resolving lipid mediators known as resolvins, lipoxins, and macrophage mediators in resolving inflammation (maresins), all of which are derived from polyunsaturated fatty acids (4, 128). Resolvins act to block the migration of neutrophils and monocytes, and can reduce the oxidative burst of neutrophils (129). Similarly to α7 nAChR-expressing cells, maresins can inhibit NF-kB activity in macrophages and help promote microglial differentiation to the M2 phenotype (130). Together, these lipid mediators represent possible new therapeutic targets for POCD. Lastly, the vagus nerve can also promote the restoration of BBB integrity *via* netrin-1, a protein involved in cell migration and axonal pathfinding during development (34), however netrin-1 has yet to be explored as a therapeutic target for POCD in human studies.

## Etiology of POCD

It is difficult to determine the etiology of POCD as surgery and anesthesia are virtually inseparable in modern society. As a result, surgery and anesthesia act as natural confounders of each other, hindering an understanding of a causal relationship and spurring controversy in the literature. Carefully designed animal and human studies have been developed to tease out the contributions of surgery or anesthesia to the development of POCD, however there is great variability in experimental design, limiting the interpretation of these results.

### Evidence From Animal Models

Animal models can provide strong insight into the etiology of POCD by exposing a genetically identical group to different anesthetic or surgical regimens and comparing the rates of POCD across groups. Moreover, animal models have the advantage of assessing neuroinflammation at the level of brain parenchyma in terminal experiments, creating a vital link to the neuroinflammatory hypothesis. Much of the evidence supports the notion that surgery and not anesthesia causes both neuroinflammation and POCD: For example, studies in rodents have shown that hippocampal pro-inflammatory cytokines IL-1β, IL-6, and TNFα (70) and HMGB1 (56) are increased with surgery and isoflurane anesthesia, but not with isoflurane anesthesia alone. Moreover, the same studies have shown a higher incidence of POCD (measured using spatial learning paradigms) with surgery and isoflurane compared to isoflurane alone. Increases in hippocampal IL-1β, and TNFα and impaired spatial learning have also been observed in carotid exploration surgery with propofol anesthesia but not propofol anesthesia alone (63), and no differences have been observed in POCD (measured by fear conditioning and spatial learning) between total intravenous anesthesia (TIVA) and volatile anesthetic (131). More convincingly, a recent study demonstrated that open abdominal surgery under local anesthesia caused increases in hippocampal IL-6, TNFα, and memory impairments (71), suggesting that anesthesia *per se* is not necessary for the production of neuroinflammation and subsequent development of POCD.

However, studies looking solely at the effects of anesthesia yield mixed conclusions, with anesthesia being implicated as either a causal or protective agent. Administration of “balanced anesthesia” (consisting of both intravenous and volatile anesthetic agents) during early postnatal life has been shown to produce neurotoxic effects in rats (132), and repeated exposure to the volatile anesthetic sevoflurane has been shown to affect the cognitive function of young, but not adult, mice (133). Similarly, repeated exposure to 5 h of isoflurane (end-tidal isoflurane = 0.7–1.5 vol %) in infant Rhesus macaque monkeys exposed on postnatal day (P)6, P9, and P12 resulted in evidence of motor and socioemotional deficits when tested 12 months later; infants that were only exposed once on P5 had no such alterations (134). In older mice, isoflurane alone has been associated with hippocampal inflammation and impairment of spatial memory (135), however in rats, isoflurane alone did not have an effect on spatial memory processes, even with repeat anesthetics (136). In contrast, in the setting of myocardial ischemia-reperfusion injury in rats, sevoflurane seems to exert a protective effect, mitigating impairments in long-term potentiation (LTP) and improving memory function (137, 138). While the discrepancies between these studies may be partially explained by the different experimental paradigms and the different metrics used to evaluate POCD, it may also be possible that anesthesia induces more subtle changes in cognitive function compared to surgery. One study showed that the combination of isoflurane and intraperitoneal ketamine alone decreased spatial memory and learning, but to a lesser degree than with combined anesthesia and surgery (139). Moreover, hippocampal pro-inflammatory cytokines were only increased with the combination of surgery and anesthesia, suggesting that if anesthesia alone can cause POCD, it may do so *via* non-inflammatory mechanisms. A summary of the findings of relevant animal studies can be found in Table 1.

**Table 1:** Selected relevant pre-clinical studies on etiology of POCD.

**Study**	**Animal model**	**Experimental model**	**Cognitive testing**	**Cellular/Molecular findings**	**Neurocognitive findings**
Cao et al. (70)	Adult (3–6 month) and aged (20–24 month) old Sprague Dawley rats	Partial hepatectomy under sevoflurane anesthesia vs. sevoflurane alone	Morris water maze	Upregulated expression of IL-1β and IL-6 on postoperative day 1 in all rats, and in aged rats until postoperative day 3	Surgery and anesthesia, but not anesthesia alone, caused impairments in latency and distance in all rats on postoperative day 1, and in aged rats until postoperative day 3
He et al. (56)	22–23 month old Sprague-Dawley rats	Splenectomy under general anesthesia vs. 2 h isoflurane anesthesia vs. naïve control	Reversal learning version of Morris water maze	Upregulation of HMGB1 and RAGE levels in surgical group BBB disruption (by TEM) in surgical group	Surgery and anesthesia, but not anesthesia alone, caused cognitive impairments from surgery to postoperative day 3
Qian et al. (139)	20–22 month old BALB/c mice	Splenectomy with isoflurane vs. isoflurane alone vs. control	Y-maze testing	Splenectomy increased hippocampal expression of IL-1β and TNFα	Splenectomy with anesthesia and anesthesia alone both impaired cognitive testing on postoperative days 1 and 3
Tasbihgou et al. (138)	Adult male Wistar rats	Deep vs. light propofol anesthesia, with and without subsequent exposure to hypoxia	Novel object recognition test	Light anesthesia group with hypoxia had lower neurogenesis, but higher BDNF and microglia-ramification	No impairment in cognitive function in either deep or light anesthesia
Walters et al. (136)	Adult Sprague-Dawley rats	Four exposures to isoflurane anesthesia (2, 2, 4, and 6 h) over 7 weeks	Fixed consecutive number, incremental repeated acquisition, progressive ratio tasks^†^	none	No deficits in any cognitive tasks after single or repeat anesthetic exposure
Wang et al. (135)	6–8 month old male C57BL/6 mice; 14 month old male C57BL/6 mice	Isoflurane vs. no anesthetic exposure	Morris water maze	Older but not younger mice had increased hippocampal expression of NLRP3^‡^	Older but not younger mice had cognitive impairment after isoflurane anesthesia compared to no anesthetic exposure
Xu et al. (71)	9 and 18 month old female C57BL/6J mice	Laparotomy under local anesthesia (no sedation) vs. sham procedure (no incision)	Fear conditioning system	Surgery increased hippocampal levels of IL-6 and TNFα in all mice, with larger increases in older mice	Cognitive deficits with surgery alone in both young and older mice
Zhang et al. (63)	4 month old male Fischer 344 rats	Right carotid exploration with propofol and buprenorphine anesthesia vs. anesthesia alone	Barnes maze Fear conditioning system	Surgery decreased cytoplasmic hippocampal NF-κB, increased IL-1β, IL-6, MMP-9	Surgery and anesthesia, but not anesthesia alone caused impairments in cognitive metrics
Zhang et al. (131)	20 month old male Fischer 344 rats	Right carotid exploration with propofol-buprenorphine anesthesia vs. isoflurane-buprenorphine anesthesia	Barnes maze Fear conditioning system	No difference in hippocampal TNFα and IL-1β expression in propofol vs. isoflurane anesthesia	Surgery caused impairments in cognitive metrics independent of anesthetic type
Zhu et al. (137)	Adult male Wistar rats	Transient coronary artery occlusion with and without sevoflurane preconditioning vs. sham operation	N/A	Coronary occlusion increased hippocampal TNFα and IL-1β mRNA expression 1–3 days postoperatively; cytokine levels attenuated by sevoflurane	Coronary occlusion inhibited LTP compared to sham operation; sevoflurane preconditioning reversed this effect on postoperative days 1 and 3

BBB, blood-brain barrier; BDNF, brain-derived neurotrophic factor; HMGB1, high mobility group box-1 protein; IL-1β, interleukin-1 beta; IL-6, interleukin 6; LTP, long-term potentiation; MMP-9, matrix metalloproteinase 9; N/A, non-applicable; NF-κB, nuclear factor-kappa B; NLRP3, NOD-like receptor protein 3 inflammasome; RAGE, receptor for advanced glycosylation end products; TEM, transmission electron microscopy; TNFα, tumor necrosis factor alpha.

† *Rats were trained to perform these tasks for at least 15 months prior to anesthetic exposure*.

‡ *NLRP3 causes maturation and secretion of cytokines IL-1β and IL-18*.

### Evidence From Human Studies

Although human studies rely on heterogeneous populations and are limited in scope by ethical considerations, it is possible to tease out the relative contributions of surgery vs. anesthesia to the development of POCD by comparing outcomes in patients undergoing different anesthetic regimens, including general anesthesia, neuraxial anesthesia, and sedation. Indeed, a large (*n* = 636) prospective observational study comparing coronary artery bypass grafting (CABG) under general anesthesia, hip replacement under spinal anesthesia, and percutaneous coronary angiography under sedation showed no difference in POCD rates between groups (7). This result was especially interesting as rates of POCD were long thought to be higher in cardiac surgery due to the inflammation associated with CPB (2, 13). These results have been supported by prospective observational studies showing no difference in POCD between spinal vs. general anesthesia for orthopedic surgery (41, 42). Moreover, a large systematic review was unable to demonstrate a clear connection between general anesthesia and POCD (140), although the majority of studies examined were underpowered and used variable methodologies. As in animal studies, it has even been proposed that volatile anesthesia may be protective in the setting of ischemic organ damage, ultimately mitigating POCD from organ ischemia (141).

Results from randomized controlled trials, while rigorous, are inconsistent and merit further investigation into the causes of POCD. As seen in observational studies, a prospective randomized clinical trial comparing the use of general vs. spinal anesthesia in extracorporeal shock wave lithotripsy showed no significant difference in the incidence of POCD defined by a neurocognitive battery (142), suggesting that surgery and not anesthesia causes POCD. In a separate study of patients undergoing CABG using high-dose vs. low-dose fentanyl anesthesia, the same group showed no difference in POCD at 3 and 12 months following surgery, although low-dose fentanyl did have higher rates of POCD at 1 week following surgery (143). However, randomized controlled trials comparing propofol and volatile anesthesia in laparoscopic cholecystectomy (144) and esophageal resection (145) have shown a higher incidence of POCD and pro-inflammatory markers with volatile anesthesia. It is important to note that these trials used different neurocognitive assessments to identify POCD, including the mini-mental status exam (MMSE) and the Montreal Cognitive Assessment (MoCA). Other randomized clinical trials attempting to show a dose-response effect with volatile anesthesia have shown that high-dose anesthetic is associated with an increased incidence of POCD (146, 147). However, these trials used the Bispectral Index™ (BIS™) as a proxy for anesthetic depth, which has been shown to be influenced by a variety of non-anesthetic factors (148–150) and is often discordant with brain activity observed under anesthesia (151); thus, BIS™ may not be an accurate representation of anesthetic depth and may limit the interpretation of these studies. A summary of the findings of relevant clinical studies can be found in Table 2.

**Table 2:** Relevant clinical studies on etiology of POCD.

**Study**	**Study Type**	**Cohort**	**Sample size (*n*)**	**Surgical procedure(s)**	**Anesthetic exposure**	**Cognitive metrics**	**Key findings**
Evered et al. (7)	Prospective observational	CABG, hip replacement: adults > 55 CA: adults > 50	636	Elective CABG, hip replacement, CA	CABG: general anesthesia Hip replacement: spinal anesthesia CA: sedation	Battery of seven neuropsychological tests	No difference in POCD rates between groups (CABG−16%; hip replacement−16%; CA−21%)
Geng et al. (144)	Prospective randomized	Adults > 60	150	Laparoscopic cholecystectomy	Propofol vs. sevoflurane vs. isoflurane anesthesia	Battery of eight neuropsychological tests	Lower POCD in propofol compared to sevoflurane or isoflurane on postoperative days 1 and 3
Hirsch et al. (42)	Prospective observational	Adults ≥ 55	10	Elective major knee surgery	Spinal anesthesia with propofol sedation and femoral nerve catheter	Word list test Verbal fluency test Digit symbol test	40% POCD on postoperative day 1; 20% POCD on postoperative day 2; 40% POCD on postoperative day 3
Hou et al. (147)	Prospective randomized	Adults ≥ 60; ASA 1-2	66	Elective total knee arthroplasty	Deep vs. light anesthesia^†^ with sevoflurane and propofol, femoral and sciatic nerve blocks	MoCA Z-score < 1.96	Higher POCD in deep (20%) compared to light (3%) anesthesia
Ji et al. (41)	Prospective observational	Adults ≥ 65	83	Elective total hip replacement	Spinal anesthesia	Digit symbol substitution testConcentration endurance testNumber connection test^‡^	POCD rate 24.6% on postoperative day 7
Qiao et al. (145)	Prospective randomized	Adults 65–75	90	Esophageal resection	Sevoflurane vs. methylprednisone and sevoflurane vs. propofol	MoCA MMSE	Higher POCD in sevoflurane group on postoperative days 1, 3, 7
Shu et al. (146)	Prospective randomized	Females 20–60	192	Gynecologic laparoscopic surgery	Sevoflurane with remifentanil, titrated to BIS^††^	MMSETrail-making test	Lower POCD in 40 ≤ BIS ≤ 50 group on postoperative day 1
Silbert et al. (142)	Prospective randomized	Adults > 55 without previous neurologic deficit	100	Extracorporeal shock wave lithotripsy	General vs. spinal anesthesia	Battery of eight neuropsychological tests	No difference in POCD rates between groups
Silbert et al. (143)	Prospective randomized	Adults > 55 without previous neurologic deficit	350	Elective CABG	High-dose vs. low-dose fentanyl anesthesia	Battery of eight neuropsychological tests	Higher POCD in low-dose fentanyl group 1 week following surgery. No difference in POCD at 3 and 12 months following surgery

*ASA, American Society of Anesthesiologists Classification Scale; BIS, Bispectral Index; CA, coronary angiography; CABG, coronary artery bypass graft; MMSE, Mini-mental Status Examination; MoCA, Montreal Cognitive Assessment*.

† *Deep vs. light anesthesia determined by BIS values 40–50 vs. 55–65, respectively. ^††^BIS values stratified to three groups: 30 ≤ BIS ≤ 40, 40 ≤ BIS ≤ 50, 50 ≤ BIS ≤ 50*.

‡ *Neurocognitive tests in this study amended to a Chinese protocol*.

## Proposed Treatments for POCD

The neuroinflammatory hypothesis provides many varied targets for candidate treatments for POCD. These treatments largely fall into one of three strategies: blocking inflammation by inhibiting inflammatory mediators (anti-inflammatory), preventing the oxidative component of inflammation (anti-oxidative), or protecting neurons during and promoting neuronal health before surgery (pro-neuronal). We present an overview of multiple candidate treatments, with a brief discussion of their hypothesized mechanisms of action and their plausibility established from pre-clinical models where appropriate. We will focus on the existing human data for each treatment, where available, including ongoing human trials from the United States National Library of Medicine (ClinicalTrials.gov), the European Union Clinical Trials Register (clinicaltrialsregister.eu), and the Australian New Zealand Clinical Trials Registry (anzctr.org.au). Please see Table 3 for a summary of clinical studies for proposed treatments. For an in-depth review of the pre-clinical and human data supporting various treatments for POCD, please see Skvarc et al. (57).

**Table 3:** Clinical studies for proposed treatments for POCD.

**Treatment**	**Published clinical studies and relevant findings**	**Registered ongoing clinical trials (if applicable)**
**ANTI-INFLAMMATORY**		
COX-2 inhibitors	Zhu et al. (152): intraoperative and postoperative parecoxib vs. placebo in total knee arthroplasty (reduction in POCD at 1 week but not 3 months postoperatively) Zhu et al. (153): 1 week of preoperative celecoxib vs. placebo in total knee arthroplasty (reduction in POCD at postoperative day 7)	none
Minocycline	none	NCT02928692: preoperative minocycline vs. no treatment in colorectal surgery
Dexamethasone	Ottens et al. (154): intraoperative dexamethasone bolus vs. no treatment during cardiac surgery (no difference in cognitive performance at 1 and 12 months postoperatively)	NCT01332812: intraoperative dexamethasone bolus vs. no treatment in general surgery
Cholinergic agents	Doraiswamy et al. (155): 12-week course of donepezil >6 months vs. no treatment after CABG (improved memory recall, no improved cognition)	NCT02419352: sugammadex vs. neostigmine/atropine at end of general anesthesia NCT02927522: donepezil vs. placebo for 7 days following general surgery
Targeted cytokine inhibition	None	None
**ANTIOXIDATIVE**		
Statins	Das et al. (156): postoperative statin vs. placebo in off-pump CABG (reduced POCD on postoperative day 6)	None
N-acetylcysteine	None	PANACEA trial ACTRN12614000411640: NAC vs. placebo twice daily for 4 days beginning on day of non-cardiac surgery
Edaravone	None	None
**PRO-NEURONAL**		
Dexmedetomidine	Li et al. (157): dexmedetomidine bolus and intraoperative infusion vs. saline in laparoscopic cholecystectomy (reduced POCD on postoperative day 1) Chen et al. (158): correlation between reduction of pro-inflammatory cytokines and reduced POCD on postoperative day 1 in general surgery	NCT02275182: intraoperative dexmetedomidine vs. placebo in general surgery NEUROPRODEX trial 2013-000823-15: intraoperative dexmetedomidine vs. placebo in cardiac and abdominal surgery NCT03480061: intraoperative dexmedetomidine bolus and postoperative infusion vs. standard sedation in cardiac surgery NCT02923128: postoperative dexmedetomidine vs. sufentanil infusion in elective non-cardiac surgery
Amantadine	None	NCT03527134: five-day postoperative amantadine vs. no treatment in general surgery
Enhancing cognitive reserve	None	NCT02747784: three-month postoperative cognitive training regimen vs. no treatment in breast/urogynecological surgery
**VARIOUS TARGETS**		
Local anesthetics	Wang et al. (159): lidocaine bolus and intraoperative infusion in CABG surgery (improved working memory, verbal associative learning compared to saline controls) Chen et al. (160): lidocaine bolus and intraoperative infusion in spinal surgery (slight improvement in cognitive function)	NCT00975910: lidocaine bolus and intraoperative infusion vs. placebo in supratentorial craniotomy NCT02848599: bupivacaine vs. morphine PCA for 72 h following general surgery
Ketamine	Hudetz et al. (161): ketamine bolus at anesthetic induction during cardiac surgery (improved memory/executive function) Nagels et al. (162): ketamine bolus and intraoperative infusion following anesthetic induction during cardiac surgery (no change in POCD compared to placebo)	NCT02892916: ketamine bolus following anesthetic induction vs. placebo in elective orthopedic surgery
Lipid mediators	none	None
Cannabinoid receptors	none	none
Melatonin	Hansen et al. (163): melatonin 6 mg/kg daily for 3 months improved sleep but had no effect on POCD in women having breast cancer surgery Fan et al. (164): melatonin 1 mg/kg daily for 6 days improved sleep and improves MMSE scores in patients undergoing hip arthroplasty	none
Turmeric	none	none
Acupuncture	Gao et al. (165): Electroacupuncture preserved MMSE scores 2 and 4 days following general surgery. Lin et al. (166): Electroacupuncture preserved MMSE scores on day 3 following intestinal surgery for cancer. (167): Electroacupuncture preserved MMSE scores in elderly patients on day 3 following colorectal surgery for cancer.	none

*CABG, coronary artery bypass graft; MMSE, Mini-Mental State Exam; NAC, N-acetylcysteine; PCA, patient-controlled analgesia*.

### Anti-inflammatory

#### COX-2 Inhibitors

The cyclooxygenase 2 (COX-2) enzyme is responsible for catalyzing the conversion of arachidonic acid to pro-inflammatory prostaglandins (68) and can increase BBB permeability (69). For these reasons, COX-2 is considered an important mediator of neuroinflammation and a potential target for treatment of POCD. Indeed, rodent models have shown that the COX-2 inhibitor parecoxib is capable of downregulating IL-1 β and TNFα expression (168); furthermore, meloxicam, a non-steroidal anti-inflammatory drug (NSAID) with relative selectivity for COX-2 has been shown to decrease short-term deficits in recognition memory following surgery (169). So far, two human trials have evaluated the efficacy of COX-2 inhibition on POCD, both in geriatric patients undergoing total knee arthroplasty (152, 153). In a trial of 134 elderly patients, parecoxib was shown to decrease pro-inflammatory markers and POCD incidence (as assessed using a neurocognitive battery) compared to placebo at 1 week, but not 3 months following surgery (152), although this negative result was largely due to improved cognitive performance in the placebo group. Similarly, a trial of 178 elderly patients showed that celecoxib reduced pro-inflammatory markers and POCD (determined by reduction in performance of ≥2 of 5 cognitive tests) at 1 week following surgery compared to placebo (153). There are no ongoing registered clinical trials testing the use of NSAIDs or selective COX-2 inhibitors in POCD.

#### Minocycline

Minocycline is a second-generation tetracycline antibiotic that has anti-inflammatory properties; it has shown to be useful in reducing cognitive deficits in animal models of cerebral ischemia, Alzheimer's disease, and Parkinson's disease (170). Minocycline readily crosses the BBB, and thus may also be useful in inhibiting neuroinflammation. In rats, minocycline has been shown to block IL-1β, with a concomitant reduction in surgery-induced hippocampal-dependent memory impairment (determined by fear conditioning test) (73). In mice undergoing appendectomy, preoperative administration of minocycline has been shown to downregulate production of IL-1β, IL-6, and TNFα, inhibit microglial activation, and impair learning (measured *via* Morris water maze and fear conditioning test) (171). However, it has been recently demonstrated in aged rats undergoing abdominal surgery that minocycline may simply delay microglial activation (172). Thus, while it has been proposed that minocycline may be useful for reducing POCD, it may not prevent development of delayed POCD. Currently, there is a multicenter randomized Phase 3 clinical trial recruiting patients to investigate the efficacy of preoperative minocycline in reducing POCD in patients with colon cancer undergoing colorectal surgery (ClinicalTrials.gov identifier NCT02928692).

#### Dexamethasone

Dexamethasone is a corticosteroid with glucocorticoid actions and powerful (>30 times more potent than cortisol) anti-inflammatory properties. As with other steroid hormones, dexamethasone inhibits the infiltration of leukocytes into the target inflammatory region (173); moreover, it can downregulate the transcription of cytokines and other cell adhesion molecules (174). Although dexamethasone has well-demonstrated anti-inflammatory actions, it is unclear whether it may have an effect on the development of POCD. In a study by Karaman et al. (175), male rats given sevoflurane were shown to develop memory deficits (measured *via* Morris water maze) at 7 and 30 days post anesthesia. Administration of 0.1 mg/kg dexamethasone before anesthetic administration reversed these deficits at both time points, suggesting its utility in mitigating POCD. However, a randomized clinical trial of patients given 1 mg/kg intraoperative dexamethasone during cardiac surgery failed to demonstrate a difference in cognitive performance both at 1 month and at 12 months following surgery (154). There is only one registered clinical trial on dexamethasone and POCD (ClinicalTrials.gov identifier NCT01332812); this Phase 4 study of 300 patients compared administration of 8 mg of dexamethasone following anesthesia induction vs. no injection and measured POCD *via* a cognitive battery up to 180 days post-surgery. Currently no results are reported.

#### Cholinergic Agents

The cholinergic anti-inflammatory pathway provides a variety of potential therapeutic targets for POCD. Both the α7 nAChR agonist PHA 586487 (176) and physostigmine (125) have been shown to reduce pro-inflammatory cytokines and neuronal damage in rat hippocampus following surgery. However, neither of these studies evaluated behavioral impairments, limiting their generalizability to POCD. In humans, during anesthetic emergence, patients are often given cholinesterase inhibitors such as neostigmine to reverse neuromuscular blocking agents (which are routinely administered to help provide optimal surgical conditions). However, the cyclic oligosaccharide sugammadex, which rapidly and profoundly reverses neuromuscular blockade by encapsulating nondepolarizing steroidal neuromuscular blocking agents such as rocuronium and vecuronium (177), has significantly reduced the use of cholinesterase inhibitors during surgery and provides a unique way to test the association of cholinesterase inhibitors on POCD. Indeed, one registered clinical trial (ClinicalTrials.gov identifier NCT02419352) has randomized 160 patients to receive either sugammadex or the combination of neostigmine and atropine at the end of surgery and anesthesia; results have not yet been published. Other human studies have focused on the anticholinesterase drug donepezil as a potential therapy. In a pilot randomized clinical trial of 44 patients, Doraiswamy et al. (155) showed that a 12-week course of donepezil given at least 6 months following CABG surgery improved memory recall but not cognition. A new Phase 3 clinical trial (ClinicalTrials.gov identifier NCT02927522) plans to randomize over 500 patients to receive donepezil or placebo for 7 days following surgery, and evaluate for POCD 1 week following surgery (although it is unclear what psychological tests are used to define POCD in this study). Again, although vagal stimulation has been proposed to mitigate the development of POCD (127), there are no current human trials designed to test this hypothesis.

#### Targeted Cytokine Inhibition

Although there are currently no human data and no registered clinical trials, drugs that block specific cytokines are already utilized as treatment for chronic inflammatory diseases such as rheumatoid arthritis (RA) and may be a potential target for POCD therapies. The IL-1 receptor antagonist anakinra represents one such target: It has been shown that IL-1 knockout mice have lower levels of IL-6 following peripheral surgery, and less memory impairment (73). Similarly, intracisternal administration of an IL-1 receptor antagonist immediately preceding abdominal surgery in aged rats prevented a decrease in memory consolidation on postoperative day 4 (178). The anti-TNFα antibody Etanercept (also used in RA) may be another target for POCD, as preoperative administration of anti-TNFα antibody inhibited IL-1β production in mice and mitigated memory impairments in mice (35). Further, the IL-6 receptor antibody toclizumab has been shown to reduce memory impairments in mice following surgery (179).

### Antioxidative

#### Statins

Statins are reversible competitive inhibitors of the enzyme 3-hydroxy-3-methylglutaryl-CoA reductase (HMG-CoA reductase). This enzyme catalyzes the conversion of HMG-CoA to mevalonate, and is the rate-limiting step of cholesterol synthesis from fatty acids (180). As part of this enzymatic process, NADPH is produced; by inhibiting HMG-CoA reductase, NADPH production is lowered, which can reduce the levels of oxidative species (181). Statins have been widely proposed to be beneficial for neurological disorders including dementia (182) and postoperative delirium (183). In POCD, a small randomized controlled trial comparing postoperative statin vs. placebo administration in patients undergoing off-pump CABG showed a significant reduction in memory dysfunction (measured by postgraduate institute memory scale) on postoperative day 6 (156). Unfortunately, no other prospective clinical trials are currently underway to investigate the otherwise promising effects of a widely utilized drug.

#### N-Acetylcysteine

N-acetylcysteine (NAC) has antioxidant properties which are related to its role as a precursor for glutathione synthesis (184). Additionally, in pre-clinical studies, NAC has been shown to downregulate pro-inflammatory cytokine synthesis including HGMB-1 (185), upregulate anti-inflammatory cytokine synthesis (186), and reduce microglial activation (187). A systematic review of the human literature has suggested that NAC supplementation can have beneficial cognitive effects for patients with a wide variety of neurological and psychiatric disorders, including Alzheimer's disease, traumatic brain injury, Parkinson's disease, and addictive behavior (184), thus raising the possibility of NAC as a potential treatment for POCD. Only one randomized controlled trial, The Post-Anesthesia N-acetylcysteine Cognitive Evaluation (PANACEA) trial (Australian New Zealand Clinical Trials Registry identifier ACTRN12614000411640) is currently being conducted to investigate the utility of NAC in POCD. This single center trial has randomized patients recovering from non-cardiac surgery to receive 1,200 mg of NAC or placebo twice daily beginning on the day of surgery and continuing for four consecutive days. POCD will be assessed *via* a neurocognitive battery at 1 week, 3, and 12 months following surgery (188). The study is ongoing and no results have been reported at this time.

#### Edaravone

Edaravone is a free radical scavenger that is used as an adjunct therapy for acute ischemic stroke in Japan, and as therapy for amyotrophic lateral sclerosis (ALS) in Japan and the United States. These uses are based on small randomized clinical trials that have shown modest efficacy in stroke (189) and early-stage ALS (190). Edaravone readily crosses the BBB, and has been shown to mitigate or ameliorate impairments in spatial and working memory in rats at 3 and 7 days following left nephrectomy and lipopolysaccharide administration (38). Moreover, the same group showed an increase in hippocampal levels of the antioxidant superoxide dismutase and a decrease in microglial activation on postoperative day 3. Taken together, this evidence suggests that edaravone has antioxidative and anti-inflammatory properties and may be a potential treatment for POCD in humans, however there are no published human studies or registered clinical trials.

### Pro-neuronal

#### Dexmedetomidine

Dexmedetomidine is a centrally-acting presynaptic α_2_ adrenergic receptor antagonist used for sedation in the operating room and intensive care unit; its mechanism of action is inhibition of norepinephrine release from adrenergic neurons projecting from the locus coeruleus to the basal forebrain, anterior cortex, intralaminar nucleus of the thalamus, and the preoptic area of the hypothalamus (191). Dexmedetomidine's sedative properties are largely believed to be due to norepinephrine inhibition in the preoptic area of the hypothalamus, an important nucleus in regulating arousal and sleep pathways. Dexmedetomidine is also hypothesized to have actions in the spinal cord, and is used as an adjunct for intraoperative analgesia (192) and the prolongation of regional nerve blockade (193). Recently, dexmedetomidine has been shown to enhance HMGB1 resolution in mice, likely *via* a vagotonic mechanism (194), suggesting that it also has downstream effects on reducing inflammation. Human studies have shown that dexmedetomidine bolus followed by infusion throughout laparoscopic cholecystectomy reduces serum pro-inflammatory cytokines and POCD (as measured *via* MMSE scores) compared to saline on postoperative day 1 (157). Moreover, Chen et al. (158) showed a correlation between the level of reduction of pro-inflammatory cytokines and POCD on postoperative day 1 (measured *via* MMSE), providing a much-needed link between cytokine levels and the severity of cognitive dysfunction. There are several registered ongoing Phase 4 clinical trials examining the efficacy of dexmedetomidine on POCD, comparing intraoperative dexmedetomidine to placebo (ClinicalTrials.gov identifier NCT02275182, NEUROPRODEX trial–EudraCT number 2013-000823-15), looking at late (12 months following surgery) POCD (ClinicalTrials.gov identifier NCT03480061), and comparing postoperative dexmedetomidine vs. sufentanil infusion (ClinicalTrials.gov identifier NCT02923128). So far, no data have been reported from these clinical trials.

#### Amantadine

Amantadine was initially marketed as an antiviral agent but was found to have dopaminergic actions which led to its use in Parkinson's disease (74). *In vivo*, amantadine has also been demonstrated to promote the production of glial cell line-derived neurotrophic factor (GDNF), an important pro-neuronal agent that promotes glial growth, protects glia, and inhibits microglial activation (195). In a rat surgical model, animals treated with intraperitoneal amantadine or intracerebroventricular GDNF showed a reduction of memory impairment compared to controls 1 day following surgery (74). Further, amantadine inhibited surgery induced neuroinflammation on postoperative day 1. In humans, there is only one randomized clinical trial in the recruitment phase investigating the use of a 5-day course of amantadine (beginning with one dose preoperatively) on POCD (ClinicalTrials.gov identifier NCT03527134).

#### Enhancing Cognitive Reserve

Poor cognitive function preoperatively is a potential risk factor for development of POCD, and pro-cognitive activities such as sleep, exercise, and education level seem to have a protective effect on POCD (34). As a result, it has been proposed that preoperative cognitive training may have a beneficial effect on reducing the incidence and severity of POCD. In rats, a cognitively stimulating environment has shown to attenuate surgery induced cognitive memory impairments (measured *via* novel object recognition test) and hippocampal cytokine increases (196). There is one registered clinical trial (REACT trial, ClinicalTrials.gov identifier NCT02747784) currently recruiting female patients with breast or urogynecological surgery for a 3-month postoperative cognitive training regimen compared to no treatment. Patients will be measured for POCD *via* a neurocognitive battery at 3 months following surgery; data from this trial are not available at this time.

### Candidate Treatments With Various Targets

#### Local Anesthetics

Local anesthetics such as lidocaine and bupivacaine work by stabilizing the open, inactive state of voltage-gated sodium channels; when injected peri-neuronally, the preferential local diffusion of local anesthetics to pain fibers produces its analgesic actions (197, 198). Because pain is a trigger for inflammatory pathways, it has been proposed that local anesthetics may reduce peripheral inflammation (and thus neuroinflammation and POCD). Despite the plausibility of this hypothesis, human data has not been convincing. Patients undergoing CABG surgery given lidocaine bolus 1.5 mg/kg and infusion of 4 mg/kg/h throughout surgery showed improvements in working memory and verbal associative learning compared to saline controls on postoperative day 9, however both groups had deficits in short-term memory, processing speed, and executive function (159). In spinal surgery, lidocaine bolus of 1 mg/kg followed by 1.5 mg/kg/h infusion showed a slight improvement in MMSE scores (160). Currently, there are two registered randomized controlled trials investigating the use of local anesthetics in preventing POCD. One Phase 2 trial of 100 patients with supratentorial craniotomy tested the efficacy of lidocaine bolus 1.5 mg/kg and infusion 2 mg/kg/h after induction of surgery until anesthetic emergence on POCD (ClinicalTrials.gov identifier NCT00975910), although no results have been published. Similarly, a small Phase 2 trial of 70 patients (currently under recruitment) is testing the use of postoperative bupivacaine vs. morphine patient-controlled analgesia for 72 h following surgery on POCD (ClinicalTrials.gov identifier NCT02848599), although the primary cognitive endpoint is MMSE scores at postoperative day 5.

#### Ketamine

Ketamine is an NMDA receptor antagonist with sedative, hypnotic, and analgesic properties; it is used as an anesthetic agent as well as an adjunct for neuropathic pain (191, 199). By virtue of its NMDA receptor antagonism, ketamine reduces glutamate transmission in the brain; coupled with its analgesic properties, ketamine has been proposed to reduce neuroinflammation (200). In pre-clinical models, ketamine seems to have differential effects on the levels of pro-inflammatory cytokines (201, 202), however ketamine has been shown to attenuate cognitive impairment in rodents (202, 203). Human data are equally unclear: one small clinical trial (*n* = 60) using a bolus of 0.5 mg/kg ketamine at the induction of cardiac surgery showed improved metrics of memory and executive function compared to control 1 week following surgery (161), however in a similar (but smaller) population, a 2.5 mg/kg ketamine bolus followed by 0.125 mg/kg infusion throughout the intraoperative period showed no change in POCD (measured by neurocognitive battery) compared to placebo at 1 or 10 weeks following surgery (162). It is unclear whether the discrepancies observed may be due to different dosing regimens, different cognitive assessments, or small sample size. There is currently a large (*n* = 900) randomized Phase 3 clinical trial (ClinicalTrials.gov identifier NCT02892916) recruiting patients undergoing elective orthopedic surgeries to receive a 0.5 mg/kg ketamine bolus following anesthetic induction with POCD assessment as a secondary outcome (determined by MoCA score) at 1 week and 3 months following surgery. Results are not available at this time.

#### Lipid Mediators (Resolvins, Lipoxins, Maresins)

As opposed to preventing the production of pro-inflammatory cytokines or oxidative species, lipid mediators such as resolvins, lipoxins, and maresins have begun to receive attention as possible resolvers of neuroinflammation (4, 129). In a rat model of CPB with deep hypothermic circulatory arrest, the resolution agonist annexin A1 was shown to (1) reduce systemic and neural pro-inflammatory cytokines due to inhibition of NF-κB, (2) inhibit microglial activation, and mitigate declines in Morris water maze performance at postoperative day 3 (204). Currently, no human trials exist on the role of these lipid mediators in POCD, although these agents may become more promising as more animal data become available.

#### Cannabinoid Receptors

Cannabinoids are a variety of substances that can modulate neurotransmitter release *via* cannabinoid receptors and regulate a variety of physiological processes including appetite, mood, and pain (205). The most widely known cannabinoid is tetrahydrocannabinol (THC), the psychoactive ingredient in plants of the genus Cannabis. Cannabinoids are known to suppress TLR-mediated inflammatory responses, and immune cells themselves can produce endogenous cannabinoids, possibly representing homeostatic mechanisms (206). In mice, the activation of cannabinoid receptor 2 (CR2) was shown to attenuate hippocampal memory impairment (*via* fear conditioning test) and decrease pro-inflammatory cytokines in the hippocampus and prefrontal cortex at 1, 3, and 7 days following tibial fracture surgery (207). Due to the controlled nature of exogenous and synthetic cannabinoids, there are no human data on the effects of cannabinoids on POCD, although this may represent a new area of study as cannabinoids are beginning to be used as therapy for a range of disorders including depression, anorexia, epilepsy, and multiple sclerosis (208, 209).

#### Melatonin

Melatonin is an endogenous hormone synthesized from L-tryptophan and secreted from the pineal gland. Its production is inhibited by 460–480 nm light in the blue portion of the electromagnetic spectrum and functions in maintaining circadian rhythms (210). Melatonin is also known to modulate production of pro- and anti-inflammatory cytokines and reduce cell adhesion molecules, and scavenge free radicals (211). In rodents, exogenous melatonin attenuates volatile (isoflurane)-induced memory impairment in adult and aged animals (212–214); this effect appears to result from improvements in the sleep-wake cycle (213, 214). Results from two published trials in human subjects offer no insight as to the efficacy of melatonin for the prevention of POCD. In the first instance, 54 women aged 30–75 years undergoing surgery for breast cancer were given 6 mg/day melatonin vs. placebo for 3 months beginning preoperatively again improved sleep-efficiency but without a discernable effect on POCD as measured using the ISPOCD test battery (163). In a more age-appropriate cohort of patients scheduled for hip arthroplasty (age > 65 years; *n* = 139), melatonin (1 mg/day taken orally beginning the day before surgery and continued for 5 days consecutively postoperatively) again improved sleep quality and appeared to preserve basic aspects of cognition as measured by the MMSE in the immediate (within 7 days) postoperative period (164); however, a lack of more appropriate neurocognitive assessments over a more extended time frame, preclude supporting melatonin as prophylaxis against POCD. There are no registered clinical trials currently investigating the use of melatonin in POCD.

#### Turmeric

Turmeric is a plant of the ginger family whose roots are boiled and ground for coloring and flavoring in many Eastern cultures. As such, it is comprised of many biological compounds with varying concentrations depending on the manufacturing method. One compound, curcumin, has been shown to have antioxidant and anti-inflammatory properties, possibly by inhibition of NF-κB (215). In “aged” male ICR mice (age 12 months) who underwent midline laparotomy, curcumin attenuated surgery-induced impairment in novel object recognition as well as spatial learning and memory (216); here, the anesthetic consisted solely of a neuroleptic anesthetic using fentanyl plus droperidol, so the relevance to current clinical practice is unclear. There are no published or open registered clinical trials currently investigating the use of turmeric or curcumin in POCD.

#### Acupuncture

Acupuncture is a well-known therapy in alternative medicine, having been developed more than 3,000 years ago in China. It is gaining popularity in the Western world and is being tested as a treatment for a variety of inflammatory disorders including asthma, carpal tunnel syndrome, and fibromyalgia (217). While little is known about acupuncture and POCD, recent evidence suggests that electroacupuncture increases hippocampal expression of α7 nAChRs, downregulates TNFα and IL-1β expression in hippocampal neurons, and can improve spatial memory at 1, 3, and 7 days following partial hepatectomy in rats (218). While there are no animal studies on acupuncture and POCD, three human studies (published in Chinese) were identified (165–167); sample sizes were 120, 124, and 83 subjects, respectively. Although subjects were randomized, the reported methods in each report raise enough concern as to render the validity of the data uncertain, thereby precluding a clear assessment as to the efficacy of the technique. There are no registered clinical trials currently investigating the use of acupuncture in POCD.

## Conclusion

POCD is a widespread phenomenon following the surgical experience and can have detrimental effects on an individual's functional status and quality of life. People with preexisting neurocognitive impairments seem to be exceptionally prone to developing POCD, and POCD may unmask such impairments even in the absence of clinical detection. A large and growing body of evidence from pre-clinical and clinical studies has implicated the roles of neuroinflammation in the pathogenesis of POCD, from peripheral injury to neuronal death and functional manifestations. However, the data are not entirely conclusive because of heterogeneities in animal models and human populations studied, as well as variability in pre-clinical and clinical assessments of POCD. While both animal and human studies demonstrate a variety of neuroinflammatory mechanisms at play in the perioperative period, the root causes of that inflammation, whether surgery, anesthesia, or even prior inflammation from sources such as infection are unknown. Data from randomized clinical trials seem to more strongly favor surgery as the main inciting factor of POCD, but again these data are not wholly consistent across populations, surgeries, and time scales. An alternative hypothesis is that the combination of surgery and anesthesia contributes to the pathogenesis of POCD: anesthesia may weaken the BBB by modulating tight junction protein expression (219) in a dose-dependent manner (220), while surgery provides the peripheral nidus for inflammation that is ultimately amplified in the CNS. Whatever the cause, neuroinflammation has been shown to be a common feature underlying many chronic and neurodegenerative diseases; a better understanding of such mechanisms may aid in improved diagnosis and treatment of a family of neurocognitive disorders.

The neuroinflammatory hypothesis has already generated a variety of potential candidates for treatment of POCD. The utility of many of these proposed treatment options have shown promising results in animal studies, however when applied to human populations, the treatment options yield more modest results. At this time, the lack of a formal definition of POCD is a critical barrier to future research; without a formal definition, the results of any one study may not be applicable to any other population than the one tested. Moreover, without a formal definition our understanding of the pathogenesis of POCD lacks generalizability to other neurodegenerative disorders that share common cellular mechanisms and clinical features. Only by standardizing our metrics and timepoints of POCD assessment will we be able to better understand the true incidence of POCD, compare the contributions of potential risk factors, and evaluate treatments across a large patient cohort (49, 221). Nevertheless, the sheer number of proposed treatments is suggestive of a growing interest in understanding POCD, and will hopefully benefit patients *via* a diverse array of therapies.

## Author Contributions

SAS drafted the manuscript. SAS and PAG contributed to the literature review, manuscript revision, and read and approved the submitted manuscript.

### Conflict of Interest Statement

The authors declare that the research was conducted in the absence of any commercial or financial relationships that could be construed as a potential conflict of interest.
